# Urachal adenocarcinoma that metastasized to breast was misinterpreted as primary breast mucinous carcinoma: A rare case report and literature review

**DOI:** 10.1097/MD.0000000000004612

**Published:** 2016-09-02

**Authors:** Xiang-Rong Zhao, Chao Gao, Yong Zhang, Lei Kong, Wei Qu, Jia Li, Yong-Sheng Gao, Yong-Hua Yu

**Affiliations:** a School of Medical and Life Sciences, Shandong Academy of Medical Sciences, Jinan University, Jinan, China; b Department of Radiation Oncology Ward 2, Shandong Cancer Hospital Affiliated to Shandong University; c Department of Radiation Oncology, Affiliated hospital of Shandong Academy of Medical Sciences; d Department of Pathology, Shandong Cancer Hospital Affiliated to Shandong University.

**Keywords:** breast, immunohistochemistry, metastasis, urachal carcinoma

## Abstract

**Background:**

The urachus is a vestigial tubular structure that connects the urinary bladder to the allantois during early embryonic development. Urachal carcinoma develops in the urachus, which is an embryological remnant of the urogenital sinus and allantois. The estimated annual incidence of urachal carcinoma in the general population is 0.01% of all cancers in adults. Moreover, urachal carcinoma accounts for 0.34% to 0.7% of all bladder carcinoma cases. And breast metastasis is extremely rarer.

**Methods and Results:**

A 42-year-old woman was admitted to our hospital with a palpable mass in the outer upper quadrant of the right breast, which was misinterpreted as a carcinoma that originated from the breast. Subsequently, she underwent surgery without any further meticulous examination. Immunohistochemistry analysis revealed positivity for CK20, Villin, and CDX-2 and negativity for CK7. After further inspection, a mass was found in the bladder dome using 18F-fluorodeoxyglucose positron emission tomography and computed tomography. The mass was surgically removed.

**Conclusion:**

Pathologic and immunohistochemical examination confirmed that the mass was urachal mucinous adenocarcinoma and mucinous adenocarcinoma to the right breast. The patient has been followed up without recurrence for 8 months.

## 1 Introduction

The urachus is a vestigial tubular structure that connects the urinary bladder to the allantois during early embryonic development.^[1]^ Urachal carcinoma develops in the urachus, which is an embryological remnant of the urogenital sinus and allantois. The estimated annual incidence of urachal carcinoma in the general population is 0.01% of all cancers in adults. Moreover, urachal carcinoma accounts for 0.34% to 0.7% of all bladder carcinoma cases.^[2]^ To date, distance metastasis has been presented in a number of organs, including the lung, brain, omentum, liver, bone, and lymph nodes.^[3–5]^ However, in reviewing the medical literature published in English, few breast metastasis cases have been previously reported. In the present study, we report a case of urachal mucinous adenocarcinoma with breast metastasis.

## 2 Case report

**Figure 1:**
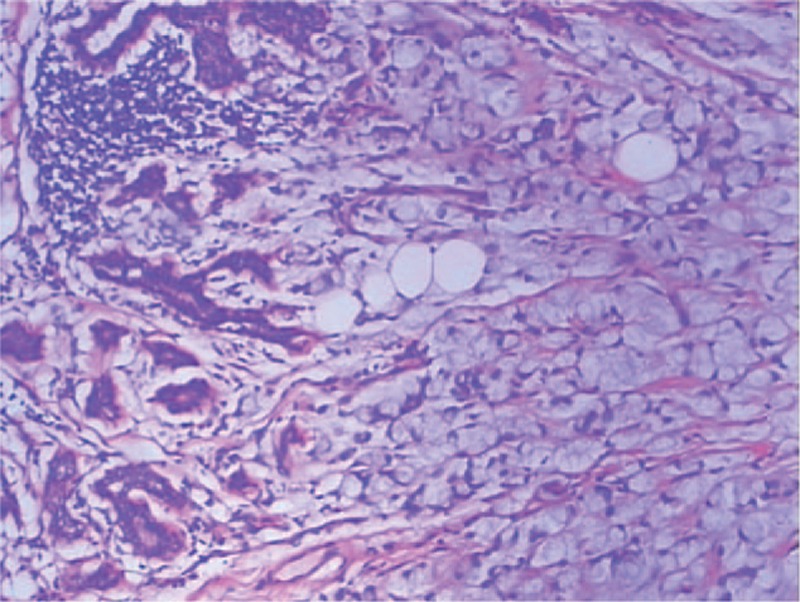
Histological examination of the metastatic breast carcinoma. The tumor specimen showed invasive adenocarcinoma, scirrhous type with formation of small ducts, and the tumor cells were surrounded by extracellular mucin (hematoxylin and eosin, ×100).

**Figure 2:**
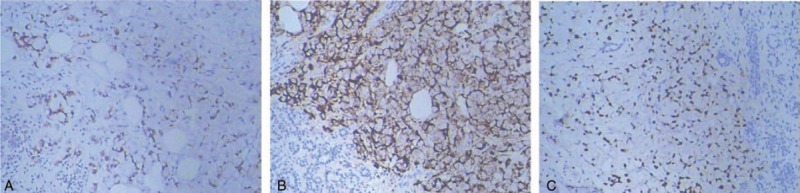
Immunohistochemical findings of the metastatic breast tumor. (A) The metastatic tumor specimen was positive expression for CK20. (B) The metastatic breast tumor specimen was positive expression for Villin. (C) The metastatic breast tumor specimen was positive expression for CDX2.

**Figure 3:**
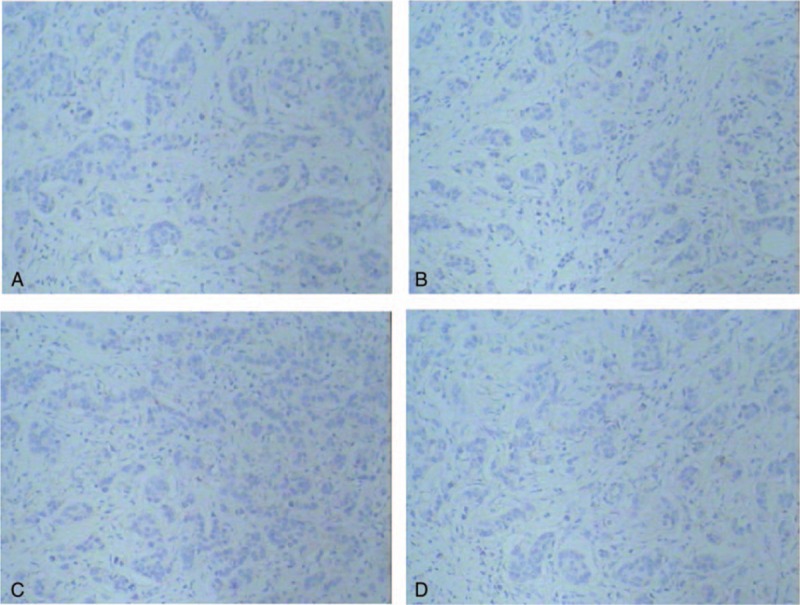
Immunohistochemical findings of the metastatic breast tumor. (A) The metastatic tumor specimen was negative expression for estrogen receptor (ER). (B) The metastatic breast tumor specimen was negative expression for progesterone receptor (PR). (C) The metastatic breast tumor specimen was negative expression for HER-2. (D) The metastatic breast tumor specimen was negative expression for GCDFP-15.

**Figure 4:**
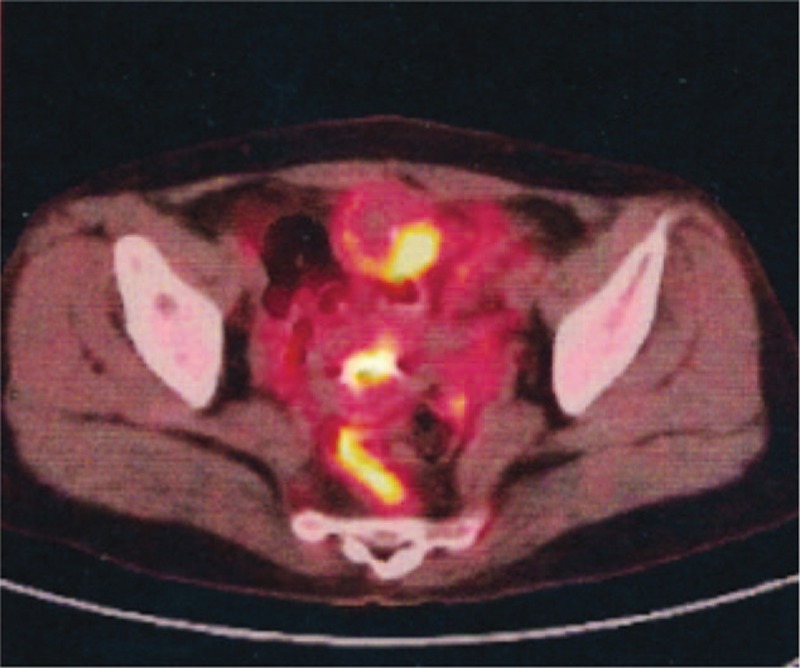
Coronal series showing space-occupying lesions in the dome of bladder combining with increased ^18^F-fluorodeoxyglucose metabolic activity unevenly: standard uptake value max 3.4.

**Figure 5:**
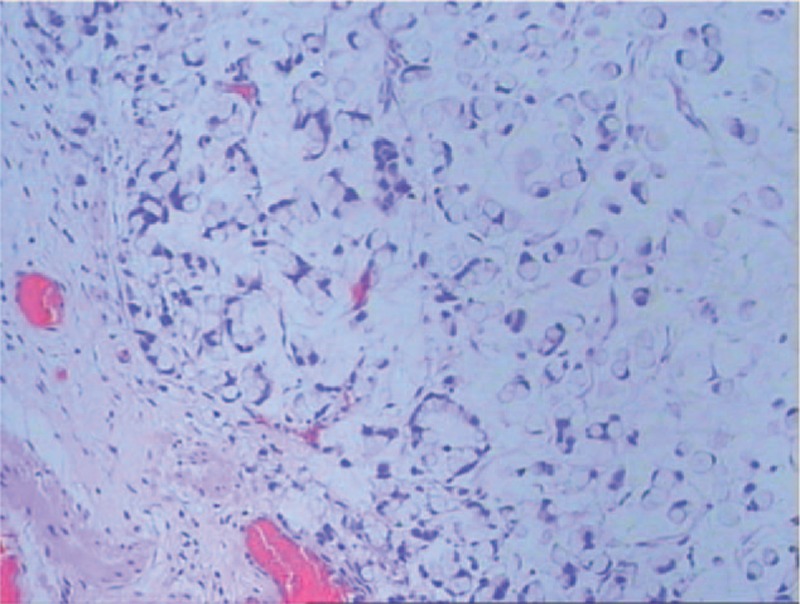
Histological examination of urachal mucinous adenocarcinoma. The tumor specimen was poorly differentiated groups of tumor cells, and the tumor cells were surrounded by extracellular mucin and partial tumor cells admixed with many signet ring cells (hematoxylin and eosin, ×100).

A 42-year-old woman was admitted to our hospital with a palpable mass in the upper outer quadrant of the right breast. The mass was tough, inactive, painless, and approximately 2.0 cm in diameter. Ultrasound examination of the right breast showed an inhomogeneous low echo. Furthermore, she had a 2-year history of urinary urgency and frequency, without hematuria, which was considered to be chronic cystitis. However, the patient did not tell the clinician about that history when she underwent urological examination until she received the subsequent positron emission tomography and computed tomography (PET/CT) results. In addition, we did not observe any abnormalities in the urine analysis. Subsequently, she underwent right breast mass resection and right modified radical mastectomy. A 2.0 × 2.0 × 1.8-cm solid lesion was found during the surgery. Intraoperative pathology revealed right breast invasive carcinoma. The postoperative course was uneventful. Histological examination of the specimen revealed right breast mucinous adenocarcinoma (Fig. 1); the ipsilateral axillary lymph nodes (0/23) were negative for metastatic carcinoma. Immunohistochemistry analysis showed positivity for CK20 (Fig. 2A), Villin (Fig. 2B), and CDX-2 (Fig. 2C) and negativity for estrogen receptor (ER) (Fig. 3A), progesterone receptor (PR) (Fig. 3B), HER-2 (Fig. 3C), GCDFP-15 (Fig. 3D). Based on these findings, we considered the lesion metastatic carcinoma. Subsequently, the patient underwent gastrointestinal examination, but a primary site was not found. Half a month after surgery, the patient was further evaluated with ^18^F-fluorodeoxyglucose (FDG)-PET/CT, which revealed no obvious residual signs in the surgical area; however, there were space-occupying lesions in the bladder dome based on the uneven increase in the ^18^F-FDG metabolic activity with an standard uptake value max of 3.4 (Fig. 4). As a result, considering the imaging and immunohistochemistry analysis, the patient appeared to have urachal carcinoma involving the partial bladder wall. Subsequently, she underwent urachal carcinoma resection with laparoscopic approach and bladder expanding excision surgery. Histology revealed a urachal mucinous adenocarcinoma, part of the signet ring cell carcinoma, with the tumor infiltrating the muscular layer as well as lymph node metastasis (1/2) in adipose tissue (Fig. 5). Finally, based on the imaging, pathology, and histology, the patient was diagnosed with urachal mucinous adenocarcinoma and mucinous adenocarcinoma to the right breast. Unfortunately, the patient rejected adjuvant therapy. The patient has been followed up without recurrence for 8 months.

## 3 Discussion

Urachal carcinoma was first described in 1863 by Hue and Jacquin in a report that was translated and summarized by Sheldon et al.^[3]^ It arises from the urachus, which is a vestigial embryonic structure located in the space of Retzius, between the bladder dome and umbilicus.^[6]^ The majority of patients are males in their fifth and sixth decades of life.^[7]^ The symptoms of urachal carcinoma commonly include hematuria, pain, umbilical discharge, and irritable symptoms.^[8,9]^

Urachal carcinoma survival tends to be poor. Ghazizadeh et al^[10]^ conducted an analysis of 66 patients with known outcomes; the overall 3- and 5-year survival rates were 13.6% and 6.0%, respectively, and the average survival of the 66 patients was 17 months. However, Ashley et al^[11]^ reported a 5-year cancer-specific survival rate of 49% in 50 years of experience at the Mayo Clinic. Moreover, the 5-year survival rate is reportedly between 16% and 45%.^[8,12]^ Possible reasons for the poor prognosis in this disease include the clinical manifestations often appear late and result in a delay in making diagnoses that are usually performed at the progressive phase, a propensity for early local invasion, and distant metastases.^[6]^

Local recurrence has been reported to occur in 50% of patients within 2 years of the original surgery,^[13]^ and the bladder, pelvis, wound, and abdominal wall are the most common sites for recurrence.^[14]^ The most commonly reported locations for distant metastasis are the lymph nodes, lungs, peritoneal cavity, anterior abdominal wall, liver, bone, brain, and ovaries, and the presence of metastasis may vary in time.^[15]^ In addition, Giordano et al^[16]^ described a patient with urachal carcinoma that metastasized to the orbit. Bastian et al^[17]^ reported a patient with metastasis from a urachal carcinoma appearing in the maxilla. Helpap and Wegner^[18]^ reported a case of urachal carcinoma with metastasis to the mandible. We report a case of urachal adenocarcinoma that metastasized to the breast, which indicated that urachal adenocarcinomas can metastasize to rare locations, such as the breast, maxilla, and mandible.

To date, there are no uniform criteria for making a diagnosis of urachal carcinoma, but many investigators agree with the MD Anderson Cancer Center criteria for diagnosing urachal carcinoma. The criteria include location in the bladder dome or elsewhere in the midline of the bladder, sharp demarcation between the tumor and normal surface epithelium, enteric type histology, absence of urothelial dysplasia, absence of cystitis cystica or cystitis glandularis transitioning to tumor, and absence of primary adenocarcinoma in another organ.^[19]^

However, these criteria are too restrictive to fulfill all prognostic criteria for a few uncommon cases. Currently, some investigators suggest 2 simpler criteria sets, which are more closely associated with clinical practice. The first set^[20]^ includes a tumor in the bladder dome, a sharp demarcation between the tumor and surface bladder epithelium, and no evidence of a primary tumor outside the bladder. The second set^[21]^ includes a tumor in the bladder dome, the presence of a residual urachal, and the absence of cystitis cystica and cystitis glandularis.

Pathologically, urachal carcinomas are usually adenocarcinomas which have a signet ring component in the form of signet ring cells combined with extravasated mucin. In addition, some urachal carcinomas have a morphology that is similar to colloid carcinomas.^[15]^ Other histologic types, such as sarcoma (leiomyosarcoma, rhabdomyosarcoma, and malignant fibrous histiocytoma), small cell carcinoma, transitional cell cancer, and mixed neoplasia, can also be found.^[10]^ In addition, they usually occur at the dome or anterior wall of the bladder.^[22,23]^ The most common histological type of urachal adenocarcinoma is mucinous adenocarcinoma.^[24]^

However, there are some different pathological features between the urachus carcinoma specimen and breast carcinoma specimen. The pathology of breast carcinoma shows uniform small round cell proliferation, arranged in cluster, floating in the lake of mucus, which is separated by fibrous connective tissue. The size and shape of the cell cluster are different, such as small tubular or papillary structure. Occasionally, cellular pleomorphism, mitotic figure, and small calcification were observed.^[25]^ Some cases were papillary and solid microcatheter in composition. Mucus stain was positive for the lake of mucus, but it was negative in the cytoplasm.^[26]^

Moreover, immunohistochemistry is one of the most important methods for differentiating between primary and metastatic carcinoma. According to previous studies, Scopsi et al^[25]^ indicated that urachal adenocarcinomas are commonly positive for CK7, CK20, and CDX2, and they lack nuclear positivity for β-catenin. Furthermore, Gopalan et al^[15]^ reported that all urachal carcinomas are diffusely and strongly positive for CK20 and CDX-2, and approximately half of urachal carcinomas are positive for CK7. However, primary breast cancers are usually positive for ER, PR, C-erbB2, Cyclin D1, and so on. Thus, it is easy to differentiate primary and metastatic breast carcinoma, as immunohistochemistry is a useful tool. In the present report, immunohistochemistry analysis showed positivity for CK20, Villin, and CDX-2, and negativity for ER, PR, HER-2, GCDFP-15, and CK7, which supported the diagnosis of urachal adenocarcinomas. PET/CT and postoperative pathology also confirmed the immunohistochemistry results. Therefore, we should perform meticulous examination of the patient before employing the next treatment strategy, and biopsy should be utilized to acquire a sample for pathology analysis and immunohistochemistry to confirm the disease.

Unlike other carcinomas, there is no standard treatment for urachal carcinoma. Surgical removal of urachal carcinoma is the most effective treatment, although the standard operation method and regional lymphadenectomy approach remain to be decided.^[27]^ Primary treatment for localized disease includes wide local excision of the urachus, umbilicus, and surrounding soft tissues combined with partial or radical cystectomy and bilateral pelvic lymphadenectomy.^[9,11,28]^

Currently, there are several effective chemotherapy regimens for treating primary and metastatic urachal adenocarcinoma. For instance, Jung et al^[29]^ reported that a 5-fluorouracil-based chemotherapy regimen could be considered for metastatic recurrent disease. Yanagihara et al^[30]^ found that 5-fluorouracil, leucovorin, and oxaliplatin are effective for treating metastatic urachal carcinoma. An additional case study from Japan describes a patient with metastatic urachal carcinoma and a history of considerable chemotherapy whose lung lesions had a marked response to irinotecan.^[31]^ Furthermore, Miyata et al^[32]^ reported that the combination of gemcitabine and cisplatin may be a useful option for treating urachal carcinoma, including recurrent carcinoma. There are limited studies about radiotherapy, and it is unclear whether patients with urachal carcinoma could benefit from radiotherapy.^[33]^ In addition, the aforementioned treatment outcome for urachal carcinoma is usually described in case reports. However, the number of cases is low, and there are insufficient evidence-based indices that can guide chemotherapy.

Urachal carcinoma is a very rare tumor with a poor prognosis. In a previous study, survival was not associated with the age, gender, race, histological grade, or partial versus complete cystectomy.^[34]^ A study from the Mayo clinic reported that staging with the tumor node metastasis system is a predictor of the outcome after surgery for urachal adenocarcinoma.^[33]^ Moreover, Ashley et al^[11]^ observed that the most important prognosis were the tumor grade and surgical margin status. If the resection margin alone is clear, another important prognostic factor is tumor staging.^[35]^ Kim et al^[36]^ found evidence that the Mayo staging system might be more effective than the Sheldon staging system. They also suggested that the tumor size might be a prognostic factor for urachal carcinoma.

## 4 Conclusion

Urachal carcinoma is a rare tumor that can metastasize to rare locations, such as in this case study of metastasis to the breast. Some rare metastatic locations are usually ignored by clinicians, resulting in misdiagnosis. Pathological and immunohistochemical examination are essential for differentiating between urachal carcinoma and other primary carcinomas. Based on this concept, clinicians must be aware of the importance of pathology and immunohistochemistry and thus perform meticulous inspections for patients before treatment to avoid misdiagnosis. Moreover, there is no standard treatment for urachal carcinoma, but surgery is the main treatment of choice. Chemotherapy for urachal carcinoma should be evaluated in further research.
