# Urachal adenocarcinoma that metastasized to breast was misinterpreted as primary breast mucinous carcinoma: A rare case report and literature review: Erratum

**DOI:** 10.1097/MD.0000000000006009

**Published:** 2017-01-20

In the article “Urachal adenocarcinoma that metastasized to breast was misinterpreted as primary breast mucinous carcinoma: A rare case report and literature review”,^[1]^ which appeared in Volume 95, Issue 35 of *Medicine*, Dr. Zhao's affiliations were originally given incorrectly. The correct affiliations are as follows:

1. School of Medicine and Life Sciences, University of Jinan-Shandong Academy of Medical Sciences, Jinan, China and
2. Department of Radiation Oncology, Shandong Cancer Hospital and Institute, Shandong Academy of Medical Sciences, Jinan, China.

All other affiliations appeared correctly in the original article.
